# Supplementary material for: A Goal-Directed Program for Wheelchair Use for Children and Young People with Cerebral Palsy in Uganda: An Explorative Intervention Study
Source: J Clin Med. 2023 Mar 16;12(6):2325. doi: 10.3390/jcm12062325 (PMC10051618; doi:10.3390/jcm12062325)
Supplement: Supplementary file 1 [file jcm-12-02325-s001.zip › jcm-2288466-supplementary.pdf]

<u>Wheelchair Follow-up</u>

Name of interviewer: Follow up date:

Patient and caregiver information

Name patient:	Phone:
Name caregiver:	Phone:
Age patient:	Village/home:
Medical condition of patient:	
Chair type:	Chair size:
Chair serial number:	

Questions should be answered by the person who has received the wheelchair or by caregiver if recipient is below 18 years of age or is not mentally aware

A: Wheelchair maintenance Questions

1. Is this the first wheelchair that the patient has received? Yes/No

2. Do you still have the wheelchair from the Walkabout foundation? Yes/No

a) If No: Why do you no longer have the chair and where is it now?

3. Has the chair needed repairs? Yes/No

a) If yes, is it done? Yes/No

b) If Yes: What parts are/were in need of repair?

c) If No: Why not?

4. Do you think the 10,000 UGX you paid for the wheelchair is affordable? Yes/No

4a) Why do you think it is affordable/not affordable?

B: In which of the following activities and how often is the chair used?

In which of the following activities and how often is the chair used? (tick the appropriate box)	Not used	Daily	Weekly	Monthly
1. Sitting inside home				
2. Sitting outside in compound				
3. Eating/feeding				
4. Moving inside the house				
5. Moving around the compound				
6. Moving around the neighborhood				

7. Shopping and errands (market)				
8. Taking part in social activities in the community (e.g. parties, groups, clubs, sports, clubs, music, art, dance)				
9. Leisure activities (sports, clubs, music, art, dance, playing with other children)				
10. Taking part in religious and spiritual gatherings and activities				
11. Transport to health center (e.g. doctor, dentist, physiotherapy)				
12. Transport to working place or school				
13. At working place or at school				
14. Transport to caregivers working place				
15. For housework				
16. For digging/garden work				

17. Describe any other activities where the chair is used and how often:

C: Wheelchair satisfaction Questions

1. What have been the advantages of getting the wheelchair? (Please probe and try to get an answer that explains more in detail what they feel and think)

2. What have been the difficulties encountered while using the wheelchair? (Please probe and try to get an answer that explains more in detail what they feel and think)
